# *Saccharomyces cerevisiae*-Derived Mannan Does Not Alter Immune Responses to* Aspergillus* Allergens

**DOI:** 10.1155/2018/3298378

**Published:** 2018-01-01

**Authors:** D. Betty Lew, Kim S. LeMessurier, Maneesha Palipane, Yanyan Lin, Amali E. Samarasinghe

**Affiliations:** ^1^Department of Pediatrics, University of Tennessee Health Science Center, Memphis, TN 38103, USA; ^2^Children's Foundation Research Institute, Memphis, TN 38103, USA

## Abstract

Severe asthma with fungal sensitization predominates in the population suffering from allergic asthma, to which there is no cure. While corticosteroids are the mainstay in current treatment, other means of controlling inflammation may be beneficial. Herein, we hypothesized that mannan from* Saccharomyces cerevisiae* would dampen the characteristics of fungal allergic asthma by altering the pulmonary immune responses. Using wild-type and transgenic mice expressing the human mannose receptor on smooth muscle cells, we explored the outcome of mannan administration during allergen exposure on the pathogenesis of fungal asthma through measurement of cardinal features of disease such as inflammation, goblet cell number, and airway hyperresponsiveness. Mannan treatment did not alter most hallmarks of allergic airways disease in wild-type mice. Transgenic mice treated with mannan during allergen exposure had an equivalent response to non-mannan-treated allergic mice except for a prominent granulocytic influx into airways and cytokine availability. Our studies suggest no role for mannan as an inflammatory regulator during fungal allergy.

## 1. Introduction

Chronic respiratory diseases affect hundreds of millions worldwide [1]. Asthma is a multifaceted syndrome with various phenotypes, of which allergic asthma is the most prevalent form and is one of the most common chronic diseases in children [2]. Exposure to environmental allergens can result in the onset of symptoms, such as wheezing, coughing, and shortness of breath, which can be controlled by steroid therapies and allergen avoidance.

The involvement of the immune system in the pathogenesis of asthma is complicated and factors such as gender, age, and allergens can alter these responses. Sophisticated mouse models of asthma that utilize clinically relevant allergens such as house dust mite, fungi, and cockroach antigens can be used to study the effects that potential therapeutics have on immunomodulation during disease [3]. Fungi, for example,* Aspergillus* and* Alternaria* species, are well-known triggers of allergic diseases [4, 5]. Fungal cell wall components interact with pattern recognition receptors (PRRs) on local innate immune cells leading to inflammation [6, 7]. Allergen avoidance strategies are difficult for patients with severe asthma with fungal sensitization (SAFS) due to the ubiquitous environmental presence of fungi. Animal models of SAFS are important as they allow investigations into the pathogenesis of difficult-to-treat allergic asthma that affects the majority of patients.

Mannan, a branched polysaccharide, is a major cell wall component of fungi including* Saccharomyces cerevisiae *[8],* Candida albicans* [9], and* Aspergillus fumigatus *[10]. Mannose receptors are primarily expressed on myeloid cells like macrophages and dendritic cells wherein stimulation enhances antigen presentation [11]. Additionally, expression of these receptors on airway smooth muscle cells [12] suggests possible roles in bronchoconstriction/bronchodilation. Other functions for mannan include goblet cell (GC) hyperplasia [13], adsorption of enteropathogens [14], and promoting a healthy gut microbiome [15]. Since airways inflammation, hyperresponsiveness, and GC hyperplasia are associated with the pathogenesis of SAFS, herein, we investigated a possible role for mannan in mitigating these characteristics when administered during allergen exposure.

## 2. Materials and Methods

### 2.1. Animals and Experimental Groups

All animal work was performed under the approval of the Institutional Animal Use and Care Committee at the University of Tennessee Health Science Center. Female BALB/c mice from Jackson Laboratories (Bar Harbor, ME) and transgenic mice bred in-house were used for studies. The human mannose receptor 2 (huMRC2) cloned from bronchial smooth muscle cells was overexpressed in a mouse using smooth muscle specific SM22*α* gene promotor. Mice overexpressing huMRC2 were backcrossed onto BALB/c mice and N10 generation mice were used for experiments [16]. Mice were housed in microisolator cages with *α*-dri bedding with free access to chow in a temperature and humidity controlled room set to a 12-hour light and dark cycle. All animal work was performed during the light cycle. Untreated mice were considered “naïve” controls, those subjected to the fungal asthma model were noted as the “asthma” controls, and those treated with mannan were noted as “MN” either with or without allergy.

### 2.2. Fungal Asthma Model

Fungal asthma was induced in mice as previously detailed [17, 18]. Briefly, mice were gradually sensitized to* Aspergillus fumigatus* antigen (Greer Laboratories, Lenoir, NC) over five weeks and exposed through inhalation route to live conidia liberated from mature fungal cultures (ATCC, strain 5233) for 10 minutes. The inhalation challenge was repeated after a two-week recovery. This allergen exposure regimen leads to the development of the hallmarks of allergic asthma (elevated airways inflammation, serum IgE, GC hyperplasia, and airway wall remodeling) as previously shown [19].

### 2.3. Mannan Treatment

Mannan from* Saccharomyces cerevisiae* was purchased from Sigma-Aldrich (St. Louis, MO) and prepared for Patented Use for Asthma Therapeutic [20]. Endotoxin level was <2 EU/mL. Mice that were in groups to receive MN treatment were given 1 mg MN in 10 *μ*L intranasally one hour prior to each fungal antigen exposure.

### 2.4. Airway Hyperresponsiveness

Mice anesthetized with 12.5 mg/kg xylazine (Akorn, Inc., Lake Forest, IL) and 87.5 mg/kg pentobarbital (Sigma-Aldrich) were surgically intubated with an 18-gauge metal cannula and attached to a computer-controlled small animal mechanical ventilator (SCIREQ, Quebec, Canada). Following baseline measurements to nebulized saline, acetyl-*β*-methylcholine chloride (Sigma) was nebulized in increasing doses to determine airway hyperresponsiveness. The peak airway resistance at each dose was recorded for each animal and the mean and standard deviation were calculated for the group at each dose.

### 2.5. Tissue Harvest

Mice were euthanized seven days after the second allergen challenge, and bronchoalveolar lavage (BAL) was performed with two 1 mL aliquots of sterile phosphate buffered saline (PBS). Samples were centrifuged, and supernatants (BAL fluid) were stored at −80°C for antibody and cytokine analyses, while the cells were used for flow cytometric analyses or cytospun onto glass slides and differentially stained for morphometric analyses. Right lung lobes were harvested, snap-frozen in liquid nitrogen, and stored at −80°C for RNA analyses. Left lung lobes were harvested and fixed* ex vivo* with 10% normal buffered formalin. Blood was collected from the thoracic cavity and centrifuged to purify serum which was stored at −80°C for antibody analyses.

### 2.6. Flow Cytometry

Cells in the BAL compartment were incubated in human gamma globulin to block Fc receptors and prevent nonspecific binding of antibodies. Cells were washed and incubated in anti-CD3*ε*-Brilliant Violet 510 (BioLegend, San Diego, CA), anti-CD4-PE-Cy5 (BioLegend), anti-CD193-Alexa Fluor 647 (BD Biosciences, San Jose, CA), anti-Ly6G-V450 (BD Biosciences), and anti-Siglec-F-PE-CF594 (BD Biosciences) on ice for 30 minutes. Controls included unstained cells, isotype controls, and single color controls. Cells were washed and fixed with stabilizing fixative (BD Biosciences) and data were acquired with a BD LSR Fortessa and analyzed with FlowJo v10.1r5 (Ashland, OR).

### 2.7. Determination of GCs in Airways

Formalin-fixed left lung lobes were embedded in paraffin and sectioned along the coronal plane at 4 *μ*M and fixed onto glass slides. Deparaffinized sections were subjected to periodic acid Schiff's stain to visualize GCs along the airways by deep magenta staining. The number of GCs per 30 epithelial cells in 10 airways at 800x magnification was recorded, and the percentage of GCs for each animal was calculated prior to the calculation of the mean and standard error for the group.

### 2.8. Quantitative PCR for Changes in Gene Expression

RNA in the frozen right lung lobes was purified using TRIzol® reagent (Thermo Fisher Scientific, Waltham, MA) as per the manufacturer's recommended protocol. One microgram of RNA was converted to cDNA with iScript™ synthesis kit (Bio-Rad, Hercules, CA), and each sample was used at a 1 : 20 dilution in duplicate in SYBR® Green based qPCR reactions with validated primer assays for* Muc5ac* and* Muc5b* from Qiagen (Hilden, Germany) using an ABI7500 thermal cycler (Foster City, CA). Data were analyzed by the 2^−ΔΔCt^ method normalized to the* Hprt* housekeeping gene.

### 2.9. Quantification of Cytokines in the BAL Fluid

The collected BAL fluid from each sample was used to determine levels of canonical T_H_1 and T_H_2 cytokines and chemokines using commercially available ELISA kits as per the manufacturer's guidelines. IL-1*β*, TNF*α*, IFN*γ*, MCP-1, IL-4, IL-5, and IL-10 kits were from BD Biosciences while CCL5 and CCL11 kits were from R&D Systems (Minneapolis, MN).

### 2.10. Statistical Analyses

Each group had at least four or more animals per group and the study was repeated independently for rigor and reproducibility. Data were represented as the mean and standard deviation unless otherwise noted. Data were analyzed by one- or two-way ANOVA with Dunn's or Tukey's multiple comparisons tests, respectively, or Mann–Whitney test, or two-way ANOVA with Sidak's multiple comparisons test. The alpha was set to 0.05 in all the tests and significance denoted by asterisks (*∗*).

## 3. Results

### 3.1. Airways Inflammation Was Reduced in Mice Treated with Mannan

**Figure 1:**
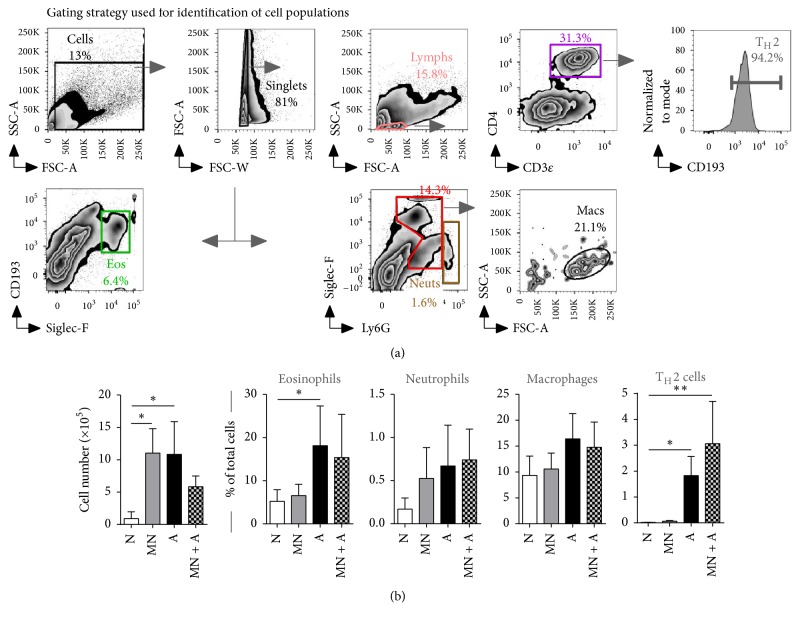
Airway inflammation after allergen exposure was reduced by MN treatment. Cells in the bronchoalveolar lavage (BAL) were analyzed by flow cytometry. Single cells expressing Siglec-F and CD193 were considered eosinophils (Eos), while Ly6G expressing cells were considered neutrophils (Neuts). Siglec-F expressing cells with high forward scatter were considered to be macrophages (Macs). CD3*ε* ^+^CD4^+^ lymphocytes (Lymphs) that expressed CD193 were considered to be T_H_2 cells (a). The total number of cells in the BAL was enumerated and the subpopulations were determined as a percentage from the total (b). MN treatment alone led to an inflammatory response in the airways but MN treatment during allergic sensitization and challenge resulted in a reduction of allergic inflammation (b). The influx of T_H_2 cells into the airways was induced in MN-treated allergic mice (b). Data are represented as the mean and standard deviation in *n* = 5–10 mice per group. Data in each group were analyzed by one-way ANOVA with Dunn's multiple comparisons test and *∗* denotes *P* < 0.05 and *∗∗* denotes *P* < 0.01.

Inflammation in the airways is a feature of asthma that is triggered when exposed to the sensitizing allergen. Our model of SAFS induces massive influx of inflammatory cells to the airways that lingers over time [19]. Cells isolated from the BAL were stained with fluorescent-labeled antibodies against markers that were used to identify specific cell populations. T_H_2 cells were identified as lymphocytes that were CD3*ε* ^+^CD4^+^CD193^+^ cells, while eosinophils were identified as cells expressing Siglec-F and CD193 (Figure 1(a)). Neutrophils were identified based on high expression of Ly6G while Siglec-F^+^Ly6G^−^ cells that have high forward scatter were considered macrophages (Figure 1(a)). Interestingly, MN treatment alone resulted in airways inflammation to equivalent levels as in those mice that were subjected to the SAFS model, although this response was repressed when MN was administered during* A. fumigatus* allergen exposure (Figure 1(b)). Eosinophils were significantly elevated in the allergic mice and MN treatment did hinder eosinophil recruitment. Neutrophils were minimal in this model and macrophage populations did not differ between the groups. T_H_2 lymphocytes were significantly increased in mice with allergy and MN treatment did not have an impact on T_H_2 cell recruitment in allergic mice (Figure 1(b)). However, these four cell types did not account for the reduction in total cells in the airways that occurred in the MN-treated allergic mice.

### 3.2. Mannan Treatment Affected Humoral Immune Responses during Allergic Asthma

**Figure 2:**
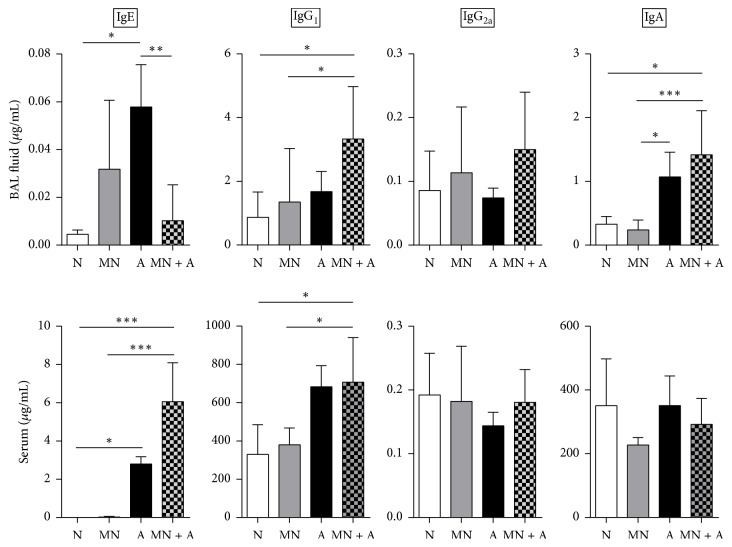
Local and systemic allergy-associated antibodies were regulated by MN treatment. Local and systemic IgE was oppositely regulated in MN-treated allergic mice wherein MN caused a reduction in the airways and an increase in the serum. Allergic mice treated with MN had increased local levels of IgG_1_, although this isotype was not altered in the serum. Levels of IgG_2a_ in the BAL fluid and serum were not affected by MN treatment while IgA was increased locally and unaffected systemically. Data are represented as the mean and standard deviation in *n* = 5–10 mice per group. Data in each group were analyzed by one-way ANOVA with Dunn's multiple comparisons test and *∗* denotes *P* < 0.05, *∗∗* denotes *P* < 0.01, and *∗∗∗* denotes *P* < 0.001.

Antibody responses are generally allergy-biased in SAFS with prominent availability of IgE and IgG_1_. Local IgE availability was reduced in MN-treated SAFS mice while serum IgE levels were unaffected by MN treatment (Figure 2). Treatment with MN also increased the amount of IgG_1_ in the BAL fluid and sera of allergic mice while IgG_2a_ remained at baseline in all groups (Figure 2). Although IgA availability in the BAL fluid increased in allergy, MN treatment did not affect its production and serum IgA levels remained at baseline in all groups (Figure 2).

### 3.3. Mannan Affected Mucus Production in Mice with SAFS

**Figure 3:**
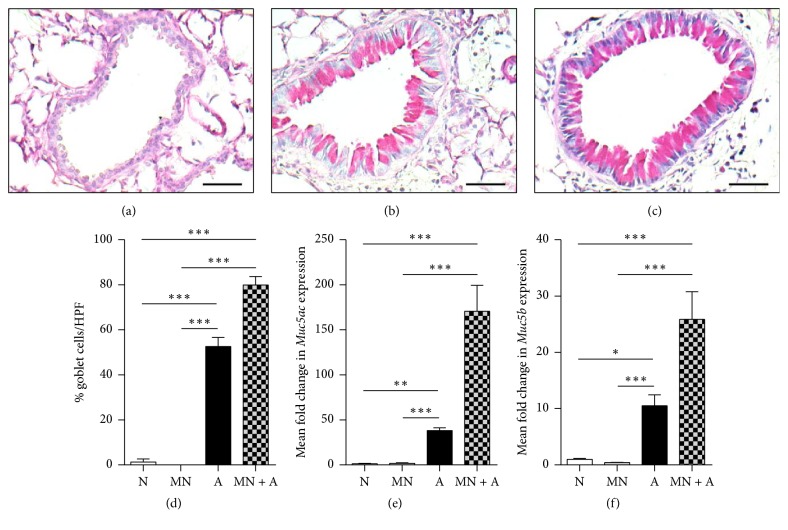
Goblet cells (GCs) were induced by MN treatment in allergic mice. While GCs were not found in the airways of naïve animals (a), GCs were abundant in allergic animals (b). MN-treated allergic mice also had GCs liberally interspersed within the airway lining (c). Enumeration of GCs per 30 epithelial cells in 10 high power fields (HPF) showed that MN treatment led to an increase in the number of GCs in allergic mice (d). This treatment also resulted in increased expression of* Muc5ac* (e) and* Muc5b* (f) in allergic mice. Data are represented as the mean and standard error in *n* = 5–10 mice per group. Data in each group were analyzed by one-way ANOVA with Dunn's multiple comparisons test, and *∗* denotes *P* < 0.05, *∗∗* denotes *P* < 0.01, and *∗∗∗* denotes *P* < 0.001.

Goblet cell hyperplasia and mucus hypersecretion are hallmarks of allergic asthma. While GCs are usually not found in naïve mouse airways (Figure 3(a)), they made up the majority of the airway lining in the SAFS groups (Figures 3(b) and 3(c)). Although there was a trend toward higher GC numbers in the MN-treated SAFS group than in the untreated SAFS mice, this increase was not statistically significant (Figure 3(d)). Expressions of mucin genes implicated in allergy,* Muc5ac* and* Muc5b*, were elevated and followed a similar trend to GC numbers (Figures 3(e) and 3(f)).

### 3.4. Mannan Treatment Had No Effect on Airway Resistance in Mice with SAFS

**Figure 4:**
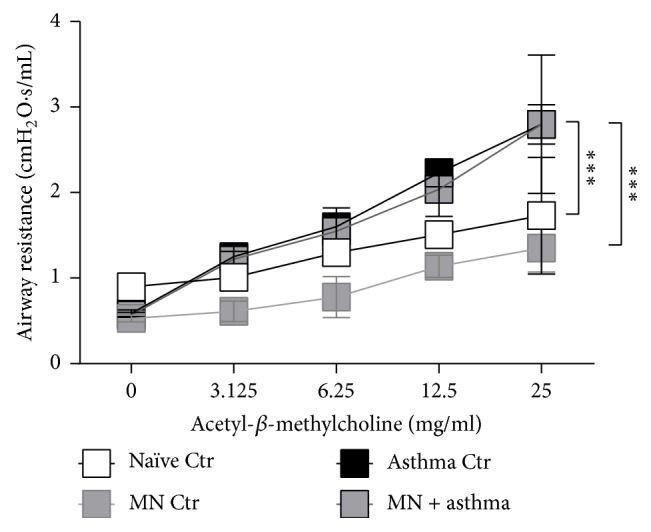
Airways resistance was unaffected by MN treatment. While naïve and nonallergic mice that received MN had no airway hyperresponsiveness, those that were sensitized and challenged with allergen had increased airways resistance. MN-treated allergic animals had an equivalent response to methacholine as allergic mice that were not treated with MN. Data are represented as the mean and standard deviation in *n* = 5–10 mice per group. Data in each group were analyzed by two-way ANOVA with Tukey's multiple comparisons test and *∗∗∗* denotes *P* < 0.001.

Airway hyperresponsiveness is a hallmark of allergic airways disease that can be measured as a response to methacholine challenge. Naïve mice and those treated with MN alone did not respond to methacholine administration (Figure 4). However, fungal allergen-sensitized and allergen-challenged mice had high airway resistance to methacholine challenge. MN-treated SAFS mice had an equivalent response, suggesting that MN does not affect airway physiologic responses (Figure 4).

### 3.5. Overexpression of huMRC2 Receptor Affected Responses to MN Treatment during Allergic Asthma

**Figure 5:**
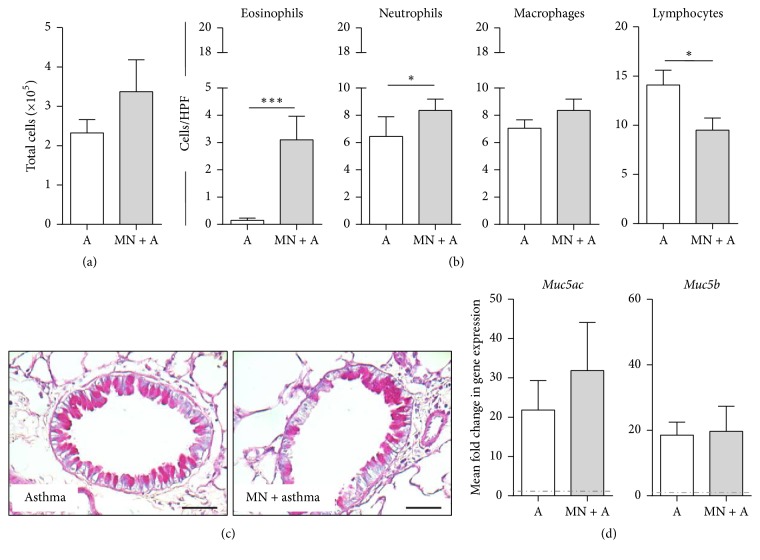
Transgenic mice expressing human MN receptor develop allergic airways disease. Airway inflammation in response to allergen sensitization and challenge in transgenic mice was equivalent with and without MN (a). However, MN treatment of these mice led to an influx of granulocytes and a reduction in lymphocytes (b). Goblet cells in the airways appeared unaffected by MN treatment (c), while expression of* Muc5ac* and* Muc5b* genes was similar between treated and untreated allergic mice (d). Data are represented as the mean and standard deviation in *n* = 4-5 mice per group. Dashed-dotted lines in (d) represent expression in naïve controls. Data in each group were analyzed by Mann–Whitney test, and *∗* denotes *P* < 0.05 and *∗∗∗* denotes *P* < 0.001.

Transgenic mice that received MN during the fungal sensitization and challenge had equivalent inflammation in the airways (Figure 5(a)). However, transgenic mice in the MN + A group had elevated numbers of eosinophils and neutrophils, similar macrophage numbers, and decreased lymphocyte population compared to transgenic allergic mice that were not treated with MN (Figure 5(b)). Transgenic naïve mice that received MN treatments did not have any response similar to WT naïve mice that were MN-treated (data not shown). MN treatment in transgenic mice resulted in a reduction in the goblet cell numbers in the airways of SAFS mice (Figure 5(c)), although mucin gene expression remained unaltered (Figure 5(d)). Interestingly, the transgenics had lower* Muc5ac* expression compared to wild-type controls (Figure 3).

### 3.6. MN Treatment Affected Airway Cytokine Availability in Transgenic Mice

**Figure 6:**
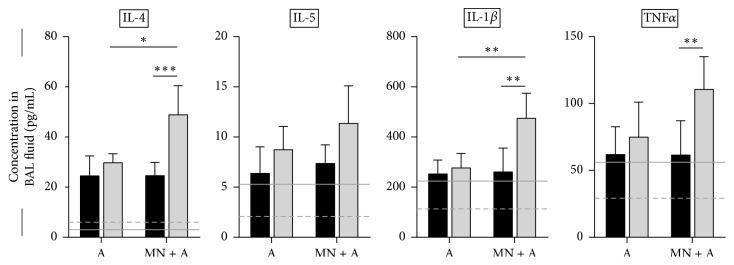
Cytokine availability in the bronchoalveolar lavage (BAL) fluid of mice subjected to the fungal asthma model with and without MN treatment. All measured cytokines were increased in the BAL fluid after allergen exposure and challenge compared to baseline levels in the naïve controls (dashed lines). Interestingly, MN treatment alone resulted in the availability of IL-5, IL-1*β*, and TNF*α* in the airways (solid lines), while IL-4 was not affected by MN treatment. While MN treatment did not have an impact on cytokine production in allergic wild-type (WT) mice, transgenic (TG) mice had a further increase in IL-4 and IL-1*β*. Cytokine levels were higher in the MN-treated TGs than WT controls. Data are represented as the mean and standard deviation in *n* = 4-5 mice per group. Data in each group were analyzed by two-way ANOVA with Sidak's multiple comparisons test. *∗* denotes *P* < 0.05, *∗∗* denotes *P* < 0.01, and *∗∗∗* denotes *P* < 0.001. Black bars denote WT mice while grey bars denote TG mice.

Cytokines in the allergic airways impact inflammation and the function of resident and recruited cells [21]. Proinflammatory and proallergic cytokines were increased over baseline levels (dashed lines in Figure 6) in allergic mice. Similar to the inflammatory response observed after MN treatment (Figure 1(b)), MN treatment alone resulted in an increase in IL-5, IL-1*β*, and TNF*α* (solid lines in Figure 6). Wild-type mice that were allergic had elevated levels of all measured cytokines, and pretreatment with MN did not affect cytokine production in these mice (Figure 6). Transgenic mice subjected to the fungal asthma model also responded with elevated cytokines (both canonical T_H_2 and T_H_1), but interestingly, MN-pretreated transgenic mice had a further increase in IL-4 and IL-1*β* compared to untreated transgenics, while this impact was not observed for IL-5 and TNF*α* (Figure 6). Additionally, MCP-1 was undetectable in the samples while MN did not affect concentrations of CCL5, CCL11, and IL-10 in either the wild-type or the transgenic mice (data not shown).

## 4. Discussion

Allergic asthma is characterized by immunologic and physiologic changes. Introduction of allergens into a sensitized lung microenvironment leads to increased inflammation, mucus production, and airway wall remodeling which can result in bronchoconstriction that manifests as breathing difficulty. Allergic inflammation, GC hyperplasia, and airway wall remodeling in our mouse model of SAFS physiologically manifest with increased airway resistance [18, 19] with close similarity to humans with severe asthma. In this study, we showed that mannan treatment does not affect most parameters of allergic responses in our SAFS model in wild-type mice. However, transgenic mice expressing huMRC2 on pulmonary smooth muscle cells had increased cytokines and granulocytes, suggesting that mannan may regulate immune functions through structural cells in the lungs.

Fungal antigens are known triggers of allergic inflammation leading to the infiltration of granulocytes into peribronchovascular areas. Components of fungal cell walls have been shown to elicit immune responses by binding to PRRs on resident cells [22]. For example,* Aspergillus* cell wall chitins induce eosinophilic inflammation [7], while fungal *β*-glucans are associated with airway epithelial cell activation and inflammation [23]. Since* A. fumigatus* is the sensitizing allergen in our model of SAFS, it is likely that a combination of fungal wall components and secreted products contribute to the development of eosinophilic inflammation. In this study, MN treatment did not alter parameters of allergic airways disease induced by* A. fumigatus *including inflammation, although it was sufficient to induce inflammation when administered alone. As such, these data may support evidence of the disconnect between physiologic and immunologic parameters during disease [24]. Since MN is also a fungal wall component, it is not surprising that MN treatment alone led to airways inflammation. The lack of regulation of responses to* A. fumigatus* may suggest that fungal components have a hierarchy/dominance in their engagement with receptors, wherein* A. fumigatus* antigen load and variation may have a dominant effect by affecting a variety of PRRs [25] while MN engages only the mannose receptors. MN binding to receptors on dendritic cells and macrophages may alter their function to favor activation. However, since MN treatment alone did not induce eosinophils or T_H_2 cells, it is possible that MN creates a bias toward T_H_1/T_H_17 responses. These possibilities warrant further studies that investigate the effect MN has on cytokine and gene signatures on myeloid cells that express mannose receptors in the context of allergy.

Stimulation of the mannose receptor on structural and immune cells of the airways can lead to increased cell activation and inflammation. Herein, we investigated the function of the mannose receptor using transgenic mice that expressed the huMRC2 on bronchial smooth muscle cells. Alterations to local immunity in transgenic mice differed from wild-type mice wherein MN treatment caused a slight increase in inflammation with a granulocytic bias recapitulating the contribution of structural cells to local immune responses. Interestingly, MN-treated transgenic mice had an increase in cytokine production compared to untreated allergen-challenged mice which may explain the increased inflammation (albeit not statistically significant). Since wild-type mice treated with MN did have a similar increase, these data also underscore the capacity of structural cells as regulators of immune responses in the lung. As part of the PRR family, mannose receptor expression typically occurs in myeloid cells that also express receptors that can bind other fungal antigens. As such, it may be of value to investigate the presence and regulation of these receptors in the context of allergen-induced inflammation, as mediators of the degree of inflammation and recruited cell types may vary depending on the types of PRRs that are activated.

Humoral immune responses characteristic of allergic airways disease are dominated by IgE, which binds to Fc*ε*RI receptors on granulocytes including mast cells, causing activation and release of mediators that can lead to vasodilation and bronchoconstriction. Antibody responses in the allergen-sensitized and allergen-challenged mice were unaltered by MN treatment, indicating that MN did not have an impact on humoral immunity. It is noteworthy, however, that MN treatment reduced the availability of IgE in the BAL fluid. Class switch recombination and IgE production occur primarily in the lymphoid organs [26, 27], but IgE can accumulate in the lungs due to increased vascular permeability during allergic inflammation. The resulting reduction of IgE in the BAL fluid in MN-treated allergic mice may be due to reduced inflammation, resident B cells class switching to produce IgE locally [28, 29], or MN treatment-induced local alterations that blunt local IgE production. Since reduction in IgE is an effective therapy to reduce asthma symptoms [30], future studies aimed at understanding the mechanism(s) by which MN treatment led to a reduction in local IgE may be advantageous.

Increased mucus production and changes in the biochemical properties of mucus are features of allergic asthma that can result in chronic cough with failure to clear sputum. Of the different types of mucins, MUC5AC and MUC5B are increased in allergic airways and these are considered markers of GC metaplasia [31]. As shown previously, goblet cell number and expression of* Muc5ac* and* Muc5b* are increased in response to fungal sensitization and challenge [18, 19]. Herein, we showed that MN treatment did not alter GC number or* Muc* genes during allergy in wild-type and in mannose receptor transgenics, suggesting that MN does not have an impact on this feature of allergy. Perhaps this is not surprising as GC hyperplasia and MUC5AC and MUC5B production are initiated by inflammatory mediators [31, 32] which were not affected by MN treatment.

## 5. Conclusions

Symptoms associated with allergic asthma can be treated, but there is currently no cure for this syndrome. While fungal wall components can result in allergic inflammation, herein, we showed that MN derived from* S. cerevisiae* did not limit features of allergic asthma induced by* A. fumigatus*. Since MN can alter the pathogenesis of ovalbumin-induced allergic airways disease [16], it is possible that this yeast component is not effective when the triggering allergen is also fungal. Further understanding on mannose receptor expression and regulation may be beneficial to explore MN as a potential therapeutic to limit allergic inflammation.
